# Surgical Resection Improves the Outcome of the Patients With Neuroendocrine Tumor Liver Metastases: Large Data From Asia

**DOI:** 10.1097/MD.0000000000000388

**Published:** 2015-01-16

**Authors:** Shunda Du, Zi Wang, Xinting Sang, Xin Lu, Yongchang Zheng, Haifeng Xu, Yiyao Xu, Tianyi Chi, Haitao Zhao, Wenze Wang, Quancai Cui, Shouxian Zhong, Jiefu Huang, Yilei Mao

**Affiliations:** From the Department of Liver Surgery (SD, ZW, XS, XL, YZ, HX, YX, TC, HZ, SZ, JH, YM); and Department of Pathology, Peking Union Medical College Hospital, Chinese Academy of Medical Sciences and PUMC, 1# Shuai-Fu-Yuan, Wang-Fu-Jing, Beijing, China (WW, QC).

## Abstract

How to properly manage neuroendocrine liver metastasis (NELM) remains debatable, and only limited clinical data have been published from Asian population. The objective of this study is to identify possible prognostic factors affecting overall survival time and to provide a guideline for future clinical practice.

A retrospective study was performed on 1286 patients who had neuroendocrine tumors in our specialized center, and data from 130 patients who had NELM were summarized. Demographic and clinicopathologic data, tumor grade, treatment method, and prognosis were statistically analyzed.

Most of the NELMs originated from pancreas (65.4%). Important prognostic factors that included tumor location and size were identified with multivariate analysis. Patients with either primary tumor resection or liver metastasis resection showed a 5-year survival of 35.7% or 33.3%, respectively, whereas resection of both resulted in a 50% 5-year survival. More importantly, resection was performed on 7 patients with grade 3 (G3) tumors, and resulted in 1-year, 3-year, and 5-year survival of 100%, 42.8%, and 28.6%, respectively, whereas the other 9 G3 patients without resection died within 3 years. *P* = 0.49 comparing the resected group with nonresected group in G3 patients. Besides, the overall 5-year survival rates for resected and nonresected patients were 40.5% and 5.4%, respectively.

Multiple prognostic factors influenced the overall outcome of NELM including patient age, tumor location, and size, etc. Aggressive surgical approaches could be considered for maximum survival time disregarding the pathological grade of the tumor. Study with larger sample size should be considered to reevaluate the recommendation of the WHO guidelines for G3 neuroendocrine tumors.

## INTRODUCTION

Gastroenteropancreatic neuroendocrine neoplasms, also referred to as neuroendocrine tumors (NETs), consist of a diverse set of rare neoplasms arising throughout the gastrointestinal tract. The incidence of NETs has increased exponentially in recent years,^1,2^ although they are regarded as rare carcinoids in general.^3,4^ The increased incidence of NETs is among the biggest in the epidemiology of neoplasms. It has become one of the most common gastrointestinal neoplasms second only to colorectal cancer.^5^

NETs remain an important clinical issue due to the high metastasis rate and the lack of an evidence-based treatment strategy.^3^ Liver is the most common place for NET distance metastasis, and literature reports showed a 46% to 93% hepatic metastasis rate.^6^ After liver metastasis (LM) has occurred, if left untreated, patients only have a 30% to 40% 5-year survival.^7,8^ Surgical management of neuroendocrine liver metastases (NELMs) remains the only therapy with the potential for a cure. It is widely accepted that surgery should be proposed in all well-differentiated NELM patients in whom complete resection is feasible irrespective of the primary tumor size and location. Various improved survival rates have been reported on resection of LM.^9–11^ However, considerable controversy exists regarding how best to manage NELM patients, with some advocating an aggressive surgical approach, whereas others adopt a more conservative strategy with more cautious approaches.^12^ There is still a lack of data on incorporating intervention toward primary tumor and hepatic metastasis together, largely due to the rarity of the patients. Most of the published results were from western countries, with only a few from Asian countries.

In this article, we reported a retrospective study of NELM with the largest patient group in Asia from a single center. The objective of the study was to identify possible prognostic factors associated with overall survival time of NELM patients, while summarizing experience in diagnosis and treatment, in hopes of providing guidelines for future clinical practice.

## MATERIALS AND METHODS

### Patients

All patient records that had been diagnosed as NETs from October 1991 to October 2013 in Peking Union Medical College (PUMC) Hospital were reviewed, and those with complete documentation and clear diagnosis of LM were included in this study. The study protocol was approved by the Ethical Committee of PUMC hospital in May 2013. Demographic and clinicopathologic data were collected, including vital status, tumor characteristics, and operative details. The diagnosis measurements, biochemical tests, treatment method, follow-up information, and survival status were summarized.

Surgical treatment included removal of the primary tumor, liver metastases, or both. All were R0 resection, whereas some patients also received postsurgical chemotherapy. Nonsurgical treatments included radiofrequency ablation (RFA), transarterial chemoembolization (TACE), targeted therapy, and chemotherapy. Postoperative follow-up was performed every 6 months or as required in the outpatient clinic. Histopathological features were reviewed including tumor diameter, presence of vascular or perineural invasion, lymph node metastasis, the number of mitosis, and percentage of Ki-67-positive cells.

### Statistical Analysis

Statistical analysis was performed using SPSS version 12.0 (SPSS Inc, Chicago, IL, USA). Data were presented as percentages, or mean or median values. Overall survival time was calculated from the date of the first treatment to the date of last follow-up or the time of death. Cumulative event rates were calculated using the method of Kaplan and Meier. Univariate analyses were performed using the log-rank test to compare differences between categorical groups. Cox proportional-hazards models were developed using relevant clinicopathologic variables to determine the association of each variable with overall survival. *P* < 0.05 was considered significant for 2-tailed probability.

## RESULTS

### Demographic and Clinicopathological Characteristics

We have reviewed all 1286 patients that had been diagnosed as NETs from October 1991 to October 2013 in Peking Union Medical College (PUMC) Hospital, and eliminated those with incomplete documentation or questionable records. A final of 130 cases were found to have liver metastases and their medical records were summarized, including 69 males and 61 females, with a mean age of 49 years ranging from 24 to 81 years’ old. The most common site for primary tumor was pancreas (65.4%), followed by stomach (10.8%) and small intestine (5.4%). Among the pancreas–liver metastases, 54.3% came from the tail, 36.6% came from the head, and 9.1% came from the uncinate process of the pancreas. The average primary tumor size was 4.2 cm (range 8–14.5 cm), with 75 patients (57.7%) having a tumor >3 cm. The average single nodule size of LM was 4.1 cm (range 3–15.0 cm), with 74 patients (56.9%) having a tumor >5 cm. Multiple NELM nodules were found in 24 cases. Sixty-eight patients underwent primary tumor resection, and 32 underwent LM resection, and 26 underwent dual resections (Table 1).

**TABLE 1:**
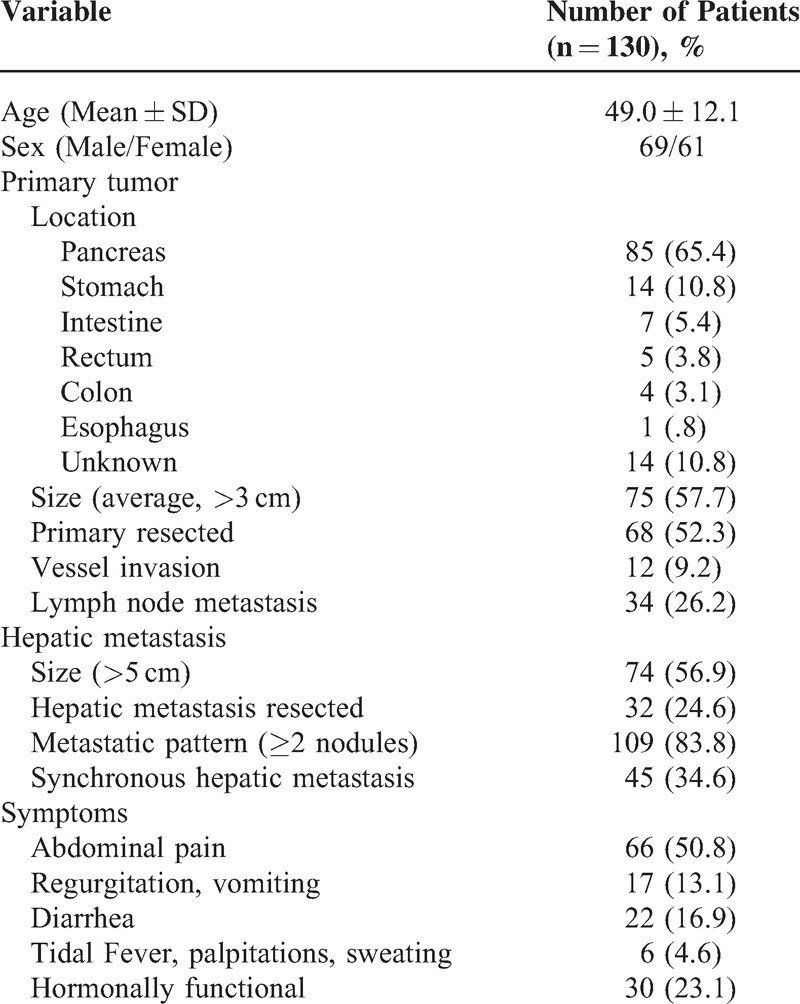
Demographic and Clinicopathological Data

### Diagnosis of the Primary NETs and LM

Twenty-five patients (19.2%) were diagnosed with NETs during routine physical examination without obvious symptoms. A total of 100 (77%) patients went to the hospital with obvious symptomatic discomfort, among whom 66 (50.8%) had abdominal pain, 17 (13.1%) had vomiting and regurgitation, 22 (16.9%) had diarrhea, and 6 (4.6%) had tidal fever, palpitations, and sweating. Thirty patients had hormone-related symptoms, presumably due to the NELMs (Table 1).

LMs were identified prior to, spontaneously, or after the diagnosis of primary NETs in 48 (38.4%), 45 (36%), and 32 (25.6%) patients, respectively. Five patients showed no record of the time of diagnosis.

Positive rate of NET detection from each imaging tool was shown in Table 2. Computed tomography (46.8%) was performed in most patients and had a positive detection rate of 46.8% (44/92), whereas B ultrasound obtained 42.2% (35/83), endoscopy 55.2% (16/29), and magnetic resonance imaging (MRI) 10% (2/20). Endoscopic ultrasonography was also used in 6 patients who had small pancreatic NETs. Digital subtraction angiography (DSA, 50%) was also employed in 6 cases and 3 were found to have NETs.

**TABLE 2:**
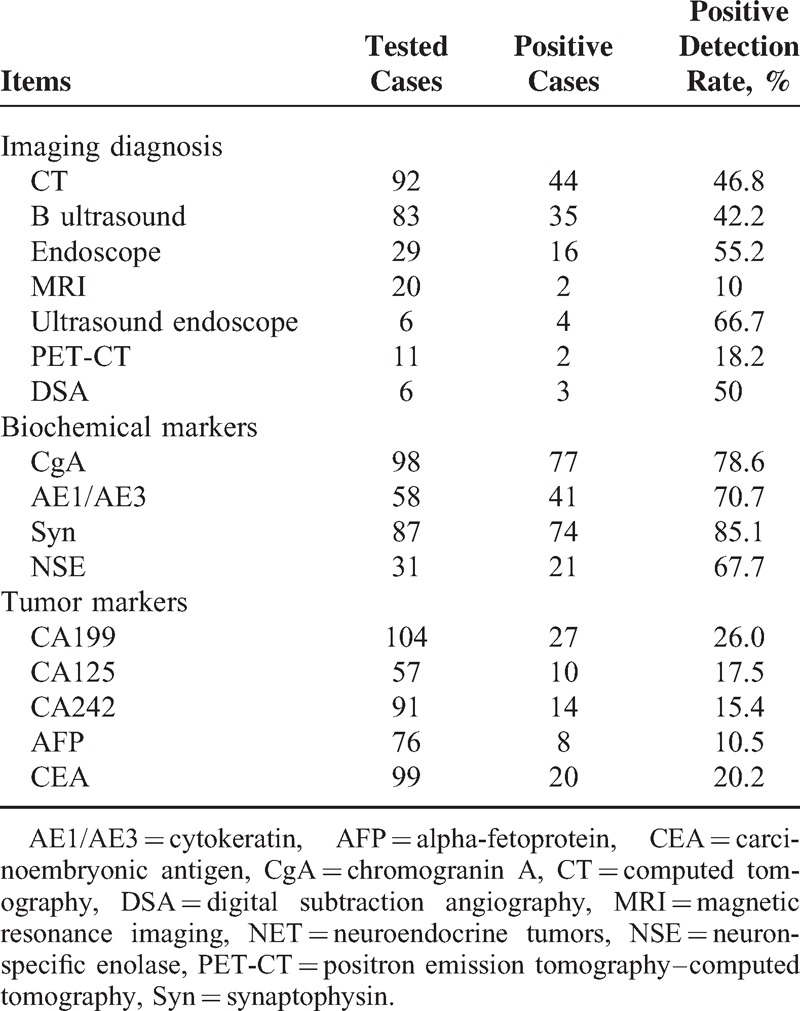
Diagnosis Measurement in NET Patients and Their Positive Detection Rates

Biochemical markers of NETs were detected by immunohistochemistry, including Chromogranin A (CgA), neuron-specific enolase (NSE), synaptophysin (Syn), and cytokeratin (AE1/AE3). The results showed that Syn had the highest positive rate (85.1 %), whereas all other markers demonstrated relatively high positive rates, including CgA (78.6%), AE1/AE3 (70.7%), and NSE (67.7%) (Table 2). Other common tumor markers were also detected as shown in Table 2, including CA199, CA125, CA242, alpha-fetoprotein and carcinoembryonic antigen; however, none of those was shown to be significantly relevant to NET diagnosis and monitoring.

### Tumor Resection and Survival Time

There are no written criteria for decision on either primary tumor resection or LM resection. In most cases, decisions were made based on the time the tumor (either primary or metastasis) being detected, the resectability of the tumor mess, and the consent of patients. The vast majority of the cases had simple excision of the primary tumor because no LM was detected before or during surgery. Three patients were operated second time to remove LM when detected later, and all others did not choose resection for various reasons, including poor physical condition, nonresectable tumor mess (6 cases), financial burden, and others. Effects of tumor resection on survival time were summarized in Table 3. Primary resection was performed on 68 patients, among whom 42 patients underwent primary resection and 26 patients underwent resection of both primary tumor and LM. Six patients underwent resection of LM only. The overall 1-, 3-, and 5-year survival rates for surgical patients were 81.5%, 38.5%, and 25.4%, respectively. The average survival time was 87.4 ± 10.2 months and 75.8 ± 12.0 months, and the 5-year survival rate was 33.3% or 35.7% for primary tumor-resected and LM-resected patients, respectively. Dual resections of both primary tumor and LM generated an average survival time of 209.1 ± 37.7 months and a 5-year survival rate of 50.0%, which were both significantly different from those of the nonresected group (*P* < 0.001). Alleviative treatment without resection of tumor only exhibited a 5.4% 5-year survival rate (Table 3). Most patients died of ruptured tumor vessels, remote metastasis, or organ failure.

**TABLE 3:**
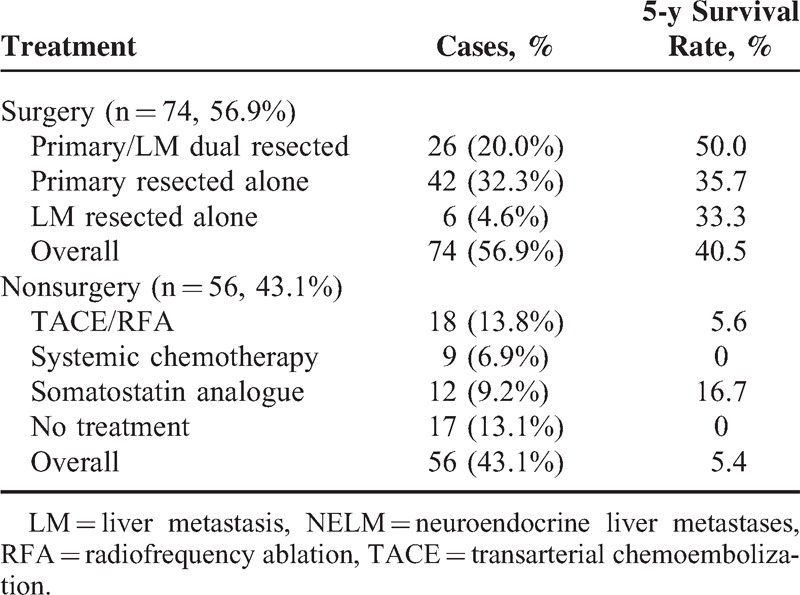
Tumor Resection on Survival Time of NELM Patients

**FIGURE 1:**
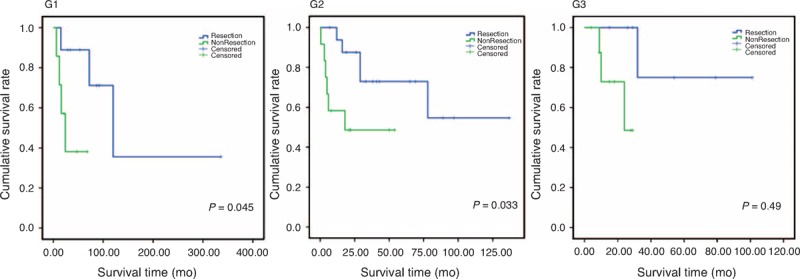
Kaplan–Meier curve upon NET grading system that showed differences in survival between resected and nonresected patients. G1, Ki67 index ≤2%; G2, Ki67 index = 2%–20%; G3, Ki67 index >20%. *P* = 0.045, 0.033, and 0.049 for G1, G2, and G3, respectively. Blue line: survival curve of patients underwent resection operation; Green line: survival curve in patients without resection of tumors. Patients with surgical resection of both primary and/or LM tumors had significantly longer survival than patients without resection in all grades. LM = liver metastasis, NET = neuroendocrine tumor.

A Ki-67 index and number of mitosis were obtained from 64 patients and these data were used to grade NETs in accordance with the WHO 2010 version of the grading system.^13^ We compared the survival time of those who undertook LM and/or primary tumor resection with those who did not, and the results were summarized in Table 4.^13^ Surgical resection brought a 5-year survival of 28.6% in G3 patients, whereas no nonresected patient survived >3 years. Significantly, increased survival time was found in all three grade levels in resected patients compared with nonresected patients, and *P* = 0.045, 0.033, and 0.049 for G1, G2, and G3, respectively (Figure 1). To further analyze the 7 G3 patients for their survival time, we found that 5 underwent primary resection (survival time: 55.0 ± 35.82 months) and 2 underwent dual resection (survival time: 30.5 ± 2.12 months).

**TABLE 4:**
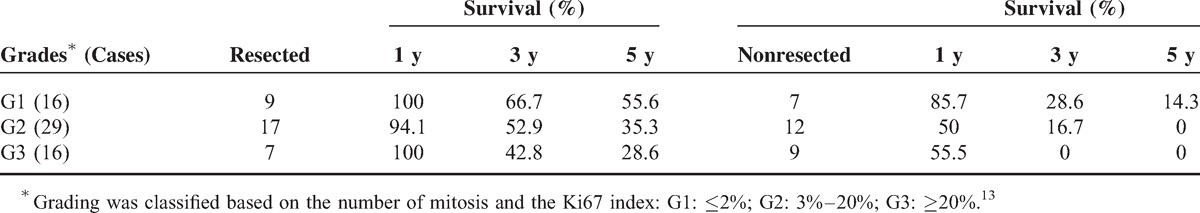
Survival at Each Grade Level Operated and Nonoperated Patients

### Other Prognostic Factors

Survival time may have been influenced by a broad range of complex factors besides management method. One of the most important factors would be systemic neo-/adjuvant chemotherapy. Three patients with advance carcinoma were treated with intervention prior to surgical resections. Ten patients were treated with systemic chemotherapy after resection (fluorouracil and/or epirubicin and/or doxorubicin and/or VP-16 and/or cisplatin, etc). Eighteen patients were treated with TACE (fluorouracil and/or epirubicin and/or doxorubicin), 6 with RFA of liver metastases patients, and 7 with long-acting somatostatin. Two patients who underwent separate resections for primary and LM tumors were treated between the two resections with either long-acting somatostatin or TACE (fluorouracil/epirubicin) therapy. However, no biostatistical analysis can be performed on these data. Besides that, by univariate and multivariate analyses, the risk factors associated with prognosis included primary tumor location (*P* = 0.03), primary tumor size (*P* = 0.01), primary tumor resection (*P* < 0.001), LM size (*P* < 0.001), and LM resection (*P* = 0.02). Vascular invasion was not included in the analysis due to incomplete data (Table 5).

**TABLE 5:**
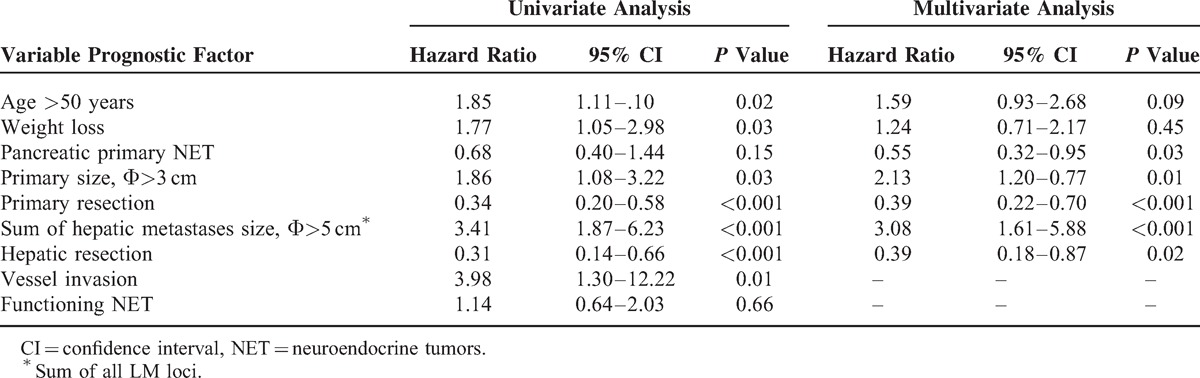
Cox Regression Analyses of Variables Associated With Survival From the Time of First Therapy Intervention

## DISCUSSION

Management of NELM may depend on factors such as tumor size, grade, location, extent of disease, tumor burden, and secretory status and potential associated symptoms.^14–18^ Surgery serves two major purposes: reducing the tumor burden and removal of excessive hormone secretion from functioning neoplasms.^19,20^ It is therefore widely regarded that resection can be beneficial to patients as long as the resection is complete, although different results have been reported.^21^ In our study, resections of both primary tumor and LM were shown to be critical for a maximum prolonged survival time, even though resection of either primary tumor or LM alone improved survival significantly compared with the nonresected group. Forty-two patients having nonresectable LM accepted primary resection alone, Pwho would typically subjected to liver transplantation,^22^ and simple resection of primary tumor received strong objections during the conference held by E-AHPBA in London 2012.^3,23^ None of our patients underwent liver transplantation, and our data proved that primary tumor resection alone was beneficial for NELM patients. Primary resection alone improved 5-year survival from 5.4% to 35.7% regardless of whether the tumor was hormonally functional. Part of the reason might rest in significant improvements in surgical technique and perioperative management that has vastly extended the application of surgical resection for these patients.

**FIGURE 2:**
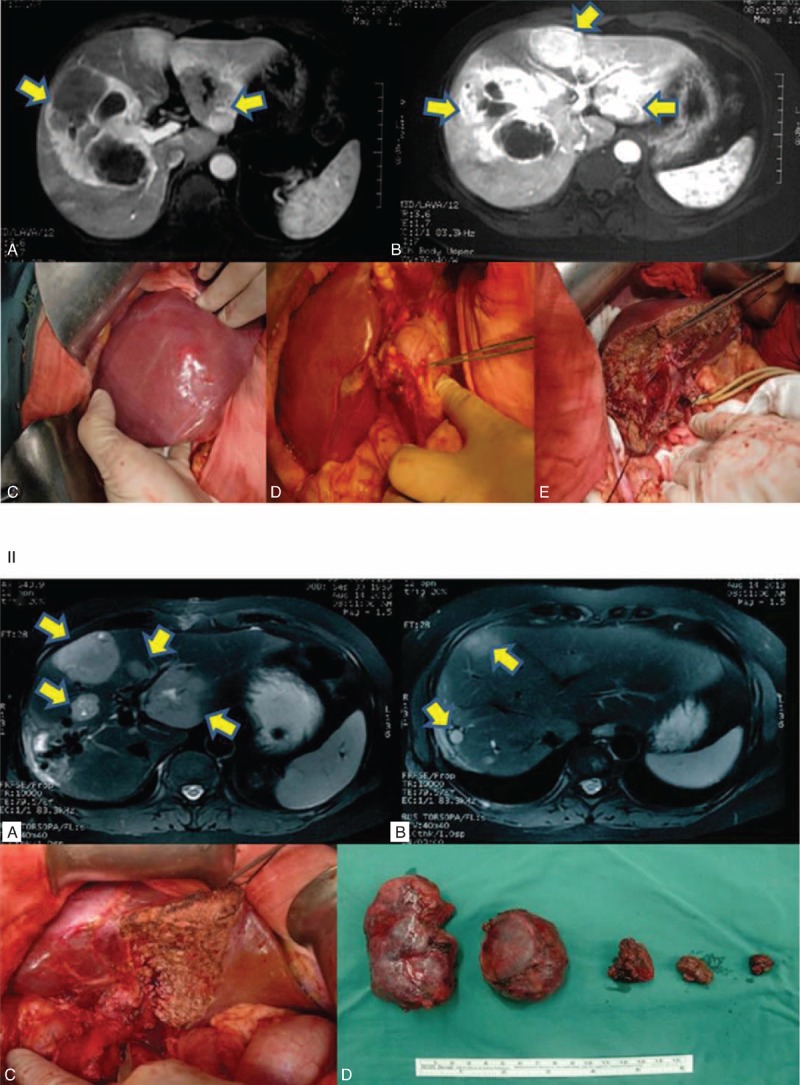
MRI and surgical data on 1 patient with G2-level NELM. A 52-year-old female patient was diagnosed with NELM with 6 liver neoplasms totaling 31 cm in size. Two separate surgical resections were performed. The first resection removed the largest tumor of 15 cm. The liver was allowed to regenerate to sufficient volume to sustain secondary resection. All 5 other neoplasms were removed during the secondary resection, which was performed 4 months after the first one. The patient was in good condition at the time of the preparation of this manuscript. Panel I, MRI images and key steps of first resection. (A & B) MRI images, arrows indicate locations of tumors; (C) a 15-cm tumor was found that covers sections of V, VI, VII, and VIII of liver; (D) primary tumor was found on the lesser curvature of the stomach; (E) remnant tissue of right lobe after resection. Panel II, MRI images and photos of second resection. (A & B) MRI images, arrows indicate locations of tumors; (C) remnant tissue after resection; (D) tumors resected from second operation. MRI = magnetic resonance imaging, NELM = neuroendocrine liver metastases

One of the important findings in our study came from the grade-level analysis. Ki67 immunostaining was performed postoperatively and the number of mitosis was calculated. None of these parameters was considered a factor at the time of surgery. However, when we compared the resected with nonresected patients at each grade level, we found that resection has been significantly beneficial to patients of all 3 grade levels. One classical case could be representative to illustrate the success of aggressive resection strategy. A patient of G2 NELM with 6 LM neoplasms totaling 31 cm in diameter underwent 2 separate resections. One tumor of 15 cm in diameter was removed through the first surgery. The liver was allowed to regenerate to sufficient volume for 4 months, and a second operation was performed to remove the remaining 5 neoplasms. The MRI data and surgical data were shown in Figure 2. The patient has remained in good condition without obvious complications for 24 months at the time of preparing this manuscript. This was a typical example that a complete resection, even nonfeasible in one surgical operation, could still be achieved through separate operations. Several other cases were successfully treated surgically, which were considered against general perception for diffused, multifocal LMs that would otherwise have been treated nonsurgically.^24^

Grading of NETs has an important role in predicting the prognosis of patients and direction of management.^25^ WHO published a classification of NETs based on the number of mitosis and the ki-67 index.^26^ Patients with poorly differentiated NET G3 (which was also called more accurately as neuroendocrine cancer) were not recommended for surgical management.^27–30^ However, it has not been validated by a prospective study, and there is a lack of data support from a broader worldwide range. We have reconfirmed Ki67 data from 64 patients, whose histological samples were available. Among 16 G3 patients, we obtained a 1-year survival of 100% in resected patients and 55.5% in nonresected patients. In this study, tumor resection significantly increased patient survival time compared with nonsurgical patients. A recently published data of nonsurgical treatment only obtained 18% in 1-year survival.^31^ Our data were the first clinical evidence demonstrating the benefit of resection on G3 grade patients since the WHO adapted the grading system in 2010. Our data provided strong indications that resection could be beneficial even on G3 grade patient, despite of the limited sample size.

We could not exclude the possible biased preselection for surgery on any late-stage neoplasm. One of the possible factors for the effectiveness of the resection would be operative techniques and personal skills. However, the reasons for patients undergoing nonsurgical procedure were multiple. Patients were denied or actively refused surgery for physical condition as well as mental and financial reasons. It is difficult to design a prospective study in which ethics for operation without pre-selection remains questionable. Retrospective study is still the only method for collecting clinical evidence for NET.

Through univariate analysis, weight loss was shown to be an influencing factor of survival at the cutoff of 2.5 kg in a 2 to 12-month period. This may need further exploration before a conclusion can be drawn, such as whether improved nutrition would have blocked weight loss and how that may affect the overall survival.^32^ Extrahepatic metastasis was reported to be correlated with poor prognosis.^33,34^ There were 40 patients having extrahepatic metastasis; however, our analysis failed to identify significant differences in survival time with or without extrahepatic metastasis.

Due to incomplete documentation or missing of record (most patients came from all over the country and had follow-ups elsewhere), we could not have an exact LM rate out of the 1286 patients. However, our reasonable estimation would be significantly lower compared with the rate in western countries (46%–93%),^6^ for the fact that all LMs would be considered for surgical resection in our hospital. The reason remained unclear. Lifestyle, environment, and genetics may all be contributing factors.^35^ Further study could be designed to pinpoint the factor(s) that influences the susceptibility to LM. Another difference was the significantly lower overall survival rates in our study compared with literature reports from western countries. One reason could be that the patient samples spun a 22-year period during which new technology and more effective methods as well as chemotherapeutic drugs had been developed rapidly. High technology and early diagnosis could have contributed to the higher survival in western countries. The second reason could be the severity of the patient when they came to our clinic. It is a general perception that most people would not see a doctor until the illness significantly affected their daily life. However, we found no differences in patients from rural areas and patients from urban areas, which suggested that accessibility to medical care was not significantly different. Ethnicity could play an important role in the outcome of survival, which was not reflected in this study.

One limitation of the study is relative small number of patient in each subgroup, given the rarity of the disease. The small sample size and heterogeneous nature of the data limited us to draw solid conclusion from the study.

## CONCLUSION

Multiple prognostic factors have been found to influence the overall outcome of NELM treatment, including age, tumor location, tumor size, etc. Being in the late stage of LM of multiple loci, patients were more apt to be rejected for surgical resection. We concluded that aggressive resection should be considered as the best effort for maximum survival time. Based on our data, the recommendation of WHO guidelines for G3 NET patients is debatable and should be reevaluated with further data.
